# Effect of Two Post-Curing Units on the Physico-Mechanical Properties of 3D-Printed Resins for Permanent Crown Fabrication

**DOI:** 10.3390/ma19091886

**Published:** 2026-05-03

**Authors:** Mazen Mujayridi, Jukka Matinlinna, Nick Silikas

**Affiliations:** 1 Division of Dentistry, School of Medical Sciences, University of Manchester, Manchester M13 9PL, UK; mazen.mujayridi@postgrad.manchester.ac.uk (M.M.); jukka.matinlinna@manchester.ac.uk (J.M.); 2 Oral and Maxillofacial Prosthodontics Department, Faculty of Dentistry, King Abdulaziz University, Jeddah 21589, Saudi Arabia

**Keywords:** 3D-printing, CAD/CAM, permanent crowns, post-curing, flexural-strength, hardness

## Abstract

Three-dimensional (3D) printing is increasingly used for the fabrication of definitive crowns; however, whether specific post-curing hardware is mandatory for clinical success remains a practical concern. This study provided a practical comparison evaluating the effect of two post-curing units on the biaxial flexural strength (BFS), Weibull modulus (m), Martens hardness (HM), indentation modulus (EIT), water sorption (WSP), and water solubility (WSL) of 3D-printed resins for permanent crowns, compared with a conventional resin composite. A total of 200 specimens were fabricated from two 3D-printed resins (Permanent Crown™ and CrownTec™) and a conventional resin composite (Filtek Universal Restorative™) used as a control. The 3D-printed specimens were post-cured using either a Formcure or an Otoflash G171 unit. WSP and WSL were measured after 90 days of water ageing, while BFS, HM, and EIT were evaluated after 24 h of storage using standardised methods. All materials exhibited WSP and WSL values within ISO limits, with the control group showing significantly higher values and superior mechanical properties. Among the 3D-printed resins, post-curing significantly affected only HM and EIT for Permanent Crown™ resin, with no significant differences in BFS. Overall, the tested 3D-printed resins demonstrated high processing stability across different curing protocols, suggesting that clinical performance remains consistent regardless of the post-curing unit used.

## 1. Introduction

Digitising the dental practice has become increasingly popular over the past few years, reflecting the growing impact of computer-aided design and computer-aided manufacturing (CAD/CAM) on modern dentistry [1]. CAD/CAM in dentistry is primarily divided into subtractive manufacturing (milling) and additive manufacturing, which is also known as rapid prototyping or 3D-printing [2]. Subtractive manufacturing has been used for decades in the dental practice, which produces dental restorations by trimming or milling the shape of the dental prosthesis from ready-made blocks or disks with great accuracy and good mechanical properties [2]. Although subtractive manufacturing is well-established in dental practice, it has different drawbacks such as the increased significant waste of the material, a time-consuming processing, especially with the complex geometries and the availability and size of the tools required to complete the process, unlike the 3D-printing, which is on the other hand, characterised by the ability to fabricate different shapes with complex geometry using variety of different materials suitable for dental application [3].

In recent years, 3D-printing in dentistry has gained significant attention due to the rapid advancement of printing technologies and their expanding applications [4]. Additive manufacturing includes several 3D-printing techniques, including stereolithography (SLA), digital light processing (DLP), selective laser melting (SLM), selective laser sintering (SLS), powder bed fusion (PBF), inkjet printing, and fused deposition modelling (FDM) [3,4]. In dentistry, the choice of 3D-printing material depends on the intended application. For example, photoactivated resins are commonly used for dental crowns and bridges, typically printed using SLA and DLP technologies.

In SLA and DLP systems, 3D-printing of polymer-based materials involves partial polymerisation during the 3D-printing process, followed by post-processing steps to ensure complete curing of the printed objects. The post-processing steps include washing the printed object in an alcohol solution to remove uncured resin, followed by post-curing under specific light and temperature conditions to achieve full polymerisation [1,3].

For optimal performance of resin-based restorations, as dental restorations are subjected to constant masticatory forces and a moisture environment with temperature and pH changes, dental resins should exhibit good mechanical properties like hardness and flexural strength, as well as good physical properties, including low water sorption (W_SP_) and water solubility (W_SL_). Water sorption is defined as the uptake of moisture from the surrounding environment, whereas water solubility refers to the leaching of unreacted or soluble components into the surrounding medium [5]. Excessive water sorption can compromise the dimensional stability of restorations, induce microcracks, and ultimately lead to clinical failure, while increased solubility may result in the release of unreacted constituents, with potential adverse effects on both material durability and biological compatibility [6].

The flexural test is commonly utilised to evaluate the mechanical strength of dental materials. Among these methods, biaxial flexural strength is considered a more reliable indicator of the long-term clinical performance of ceramic restorations. As intraoral restorations are subjected to multidirectional forces, the biaxial flexure test is considered more representative of clinical conditions than uniaxial flexural testing [7,8]. Moreover, Martens hardness is an instrumented indentation test that reflects both elastic and plastic deformation and is less influenced by the material’s viscoelastic properties, making it a potentially useful method for evaluating dental materials [9,10].

Manufacturers recommend specific post-curing steps, parameters, and devices to complete the polymerisation process, but available post-curing units differ in their light-emission technologies, such as ultraviolet (UV), light-emitting diode (LED), and flashlamp systems, each with distinct wavelength spectra. Some units even offer adjustable exposure times, temperatures, or flash frequencies, highlighting the need to optimise post-curing conditions to achieve superior mechanical properties and a higher degree of resin conversion.

Optimisation of post-curing parameters, such as curing time, temperature, and ultraviolet wavelength, is essential for improving the physical and mechanical properties and biocompatibility of 3D-printed resins [1,11]. Bayarsaikhan et al. found that increasing post-curing time and temperature improved the mechanical strength of denture base resins [2]. Additionally, Kang et al. reported that higher light intensity during post-curing enhanced the mechanical performance of interim dental resins [12]. Similarly, Aktug et al. observed that different post-curing units and exposure times affected the properties of 3D-printed resins for permanent crowns fabrication [13]. Recent studies have further demonstrated that post-curing conditions can influence the degree of conversion and trueness of 3D-printed resin materials [14,15]. Therefore, as the majority of 3D-printed dental restorations are photosensitive resins and complete polymerisation during post-curing is essential for optimal performance, further investigation into the influence of different post-curing units is necessary. This study aimed to evaluate the mechanical (biaxial flexural strength and Martens parameters, including Martens hardness (H_M_), and indentation modulus (E_IT_) and physical (water sorption and water solubility) properties of two 3D-printed resins indicated for permanent restorations, using two different post-curing units with distinct technologies.

The null hypothesis was that there would be no significant differences either between the control and the 3D-printed groups or between the post-curing units in terms of Martens hardness, indentation modulus, biaxial flexural strength, water sorption, and water solubility.

## 2. Materials and Methods

### 2.1. Study Design

**Figure 1:**
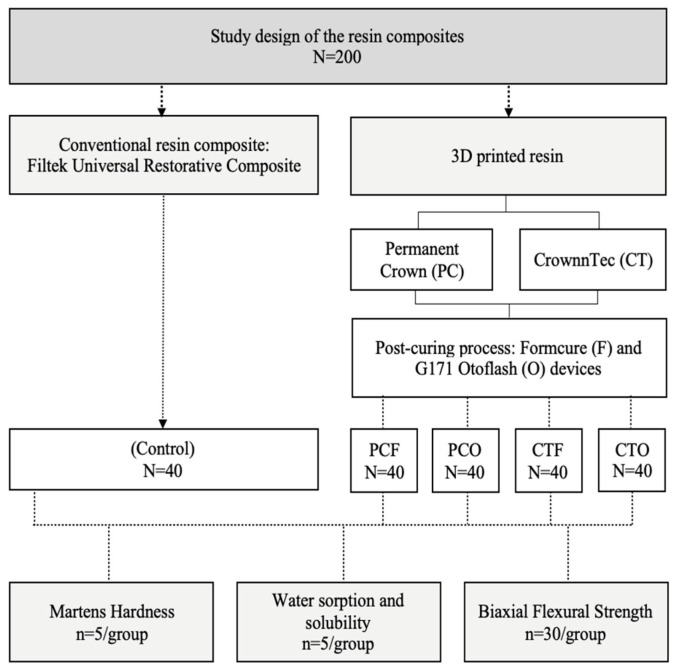
Flowchart of the experimental workflow. The study includes five groups: conventional resin composite (control) and the 3D resins groups: PCF, Permanent Crown post-cured using Formcure; CTF, Crowntec post-cured using Formcure; PCO, Permanent Crown post-cured using Otoflash; CTO, Crowntec post-cured using Otoflash. Marten’s hardness, water sorption and solubility, and biaxial flexural strength were analysed for all groups.

In this experimental study, two 3D-printable resins indicated for the fabrication of permanent restorations were used and post-polymerised in two different post-curing units following the 3D-printing and compared them to one conventional resin composite in terms of Marten’s hardness, water sorption and solubility and biaxial flexural strength. The experimental workflow of this study is illustrated in Figure 1 and Table 1.

### 2.2. Specimen Preparation

For the water sorption (W_SP_) and water solubility (W_SL_) tests, disc-shaped specimens with a diameter of 15 mm and a thickness of 1.0 mm were fabricated in accordance with ISO 10477:2020 specifications [16]. For the Martens hardness evaluation, disc-shaped specimens with a diameter of 15 mm and a thickness of 1.5 mm were prepared. Additionally, for the biaxial flexural strength assessment, square-shaped specimens with dimensions of 12 mm × 12 mm × 1.5 mm were produced.

#### 2.2.1. 3D-Printed Resins

##### Permanent CrownTM (PC) Resin

For the preparation of specimens using the Permanent Crown™ (PC) resin (Formlabs, USA), an STL file was designed using free CAD software (Tinkercad, Autodesk, San Rafael, CA, USA). Following the design step, the STL file was imported into the PreForm software (Formlabs, USA) and printed using a 3D-printer (Form 3B+, Formlabs, USA) employing stereolithography (SLA) technology. The specimens were printed with a layer thickness of 50 μm, a build orientation of 90°, and automatically generated support structures using the “mini raft” configuration (see Table 2).

After printing, the specimens were removed from the build platform and washed in 99.8% ethanol using an ultrasonic cleaning device (Form Wash, Formlabs, USA) for 5 min to eliminate uncured resin from their surfaces. The washed specimens were then left to dry at room temperature for 5 min before post-curing. Subsequently, the specimens for each experiment were randomly divided and post-cured using two different post-curing units, forming two subgroups: PCF, specimens post-cured using the Form Cure unit (Formlabs, USA), and PCO, specimens post-cured using the Otoflash G171 unit (NK-Optik, Germany), following the post-curing protocols presented in Table 3.

##### CrownTecTM (CT) Resin

The specimens of the CrownTec™ resin (Saremco Dental AG, Switzerland) were prepared by importing the STL file, designed using free CAD software (Tinkercad, Autodesk, San Rafael, CA, USA), into the 3D printer software (Composer, Asiga, Australia). The specimens were then printed using a DLP 3D-printer (Asiga Max, Australia) with a 90° build orientation, a layer thickness of 50 μm, and automatically generated support structures using the default settings (see Table 2).

After printing, all specimens were removed from the build platform and washed in 99.8% ethanol for 5 min using an ultrasonic cleaning device (Form Wash, Formlabs, USA) to remove any uncured resin from their surfaces. The washed specimens were subsequently left to air-dry at room temperature for 5 min. Following the same procedure as for the PC resin, the specimens of each experiment were divided into two subgroups according to the post-curing unit used: CTF, specimens post-cured using the Form Cure unit (Formlabs, USA), and CTO, specimens post-cured using the Otoflash G171 unit (NK-Optik, Germany), based on the post-curing parameters outlined in Table 3.

#### 2.2.2. Filtek Universal RestorativeTM (Control)

For the preparation of the control group specimens, 3D-designed moulds were created using free CAD software (Tinkercad, Autodesk, San Rafael, CA, USA). The STL files of these moulds were then imported into the 3D printer and fabricated as previously described for the 3D-printed groups. These moulds were used to prepare the control specimens for the water sorption and solubility, Martens hardness, and biaxial flexural strength tests. For the ring-shaped moulds, different thicknesses were used according to the respective tests: 1.0 mm for the water sorption and solubility tests, and 1.5 mm for the Martens hardness test. While the biaxial flexural strength test, a square-shaped mould with 12 × 12 × 1.5 mm was used. Inside each mould a separating medium was applied to facilitate the removal of the specimen, then each was filled with the conventional resin composite, tightly pressed between two microscopic glass slides, and polymerised using overlapping irradiation (multiple sites to cover all specimen surface by the light curing unit head) on one surface with a direct touch of the LED light-curing unit (Elipar™ S10, 3M, USA) at the microscopic slide surface with an intensity of 1200 mW/cm^2^ for 10 s, following the manufacturer’s recommendations.

### 2.3. Water Sorption and Solubility

Twenty-five disc-shaped specimens with a diameter of 15 mm and a thickness of 1.0 mm (n = 5 per group) were used to measure water sorption (WSP) and water solubility (WSL) in accordance with ISO 10477:2020 standards [16]. All specimens were polished using 500-grit silicon carbide abrasive paper (Buehler, Lake Bluff, IL, USA) to remove surface irregularities and ensure a standardised finish.

Following polishing, the specimens from each group were placed individually in a desiccator containing freshly dried silica gel and incubated at 37 °C for 22 ± 2 h. Subsequently, all specimens were transferred to a second desiccator at room temperature (23 ± 2 °C) for 10 min. Each specimen was then weighed periodically using an analytical balance (BM-252, A&D, Tokyo, Japan) until a constant mass (variation ≤ 0.1 mg within 24 ± 2 h) was achieved. This mass was recorded as M1.

Afterwards, each specimen was immersed individually in 10 mL of distilled water (replaced weekly) and stored in an incubator at 37 °C. At predetermined time intervals (24 h, 7 days, 14 days, 30 days, 60 days, and 90 days), the specimens were carefully removed, gently swabbed with a dry napkin, and air-dried for 15 s to remove surface moisture. Each specimen was then weighed, and the recorded mass at each time interval was designated as M(t), while the final mass after 90 days of immersion was recorded as M2.

Following the 90-day immersion period, the specimens were reconditioned individually in a desiccator containing freshly dried silica gel, using the same drying procedure as described previously, until a constant mass (variation ≤ 0.1 mg within 24 h) was reached. This final mass was recorded as M3.

To determine the mass change (%), water sorption (Wsp) (μg/mm^3^), and water solubility (Wsl) (μg/mm^3^), the following equations were applied [17]:

(1) $$Mass\;change\left(\%\right)=\frac{M\left(t\right)-M1}{M1}\times 100$$

(2) $$W_{sp}=\frac{M2-M3}{V}$$

(3) $$W_{sp}\%=\frac{M2-M3}{M1}\times 100$$

(4) $$W_{sl}=\frac{M1-M3}{V}$$

(5) $$W_{sl}\%=\frac{M1-M3}{M1}\times 100$$

where M(t) is the mass of the specimen at different time intervals, M1 is the mass of the dried specimen, M2 is the mass of the specimen after 90 days of water immersion, M3 is the mass of the reconditioned (redried) specimen after water immersion, and V is the specimen volume in mm^3^.

To determine the volume of each specimen, the diameter was calculated as the mean of 360 individual diametral readings obtained using a laser scan micrometre (LSM-6200, Mitutoyo, Japan). The thickness of each specimen was measured as the mean of three measurements taken at different locations using a digital micrometre. The specimen volume (*V*) in cubic millimetres (mm^3^) was then calculated using the following formula:

(6) $$V=\pi r^{2}h$$

where π is 3.14, *r* is the radius of the cross-section of the specimen, and *h* is the thickness of the specimen.

### 2.4. Biaxial Flexural Strength

The biaxial flexural strength was evaluated using 150 square-shaped specimens (n = 30 per group) with dimensions of 12 mm × 12 mm × 1.5 mm (± 0.05 mm). All specimens, including the control group, were polished using 500-grit silicon carbide abrasive paper (Buehler, Lake Bluff, USA) to remove any residual flanges after support structure removal and to standardise the surfaces of all samples. The specimens were then stored in distilled water at 37 °C for 24 h before testing.

**Figure 2:**
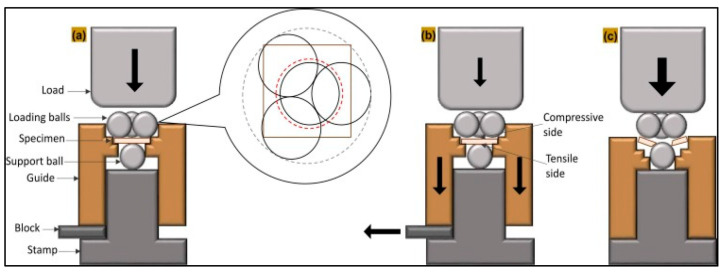
(**a**) Schematic of the B3B flexural strength test, illustrating the support ball radius (red dashed line) and the loading ball radius (black solid line). (**b**) The support guide or block was removed, allowing for free-rolling of the balls before performing the test. (**c**) Application of compressive load until fracture to record the flexural strength [18].

Following storage, the specimens were subjected to a biaxial flexural strength test using a universal testing machine (Zwick Roell Z020, Ulm-Einsingen, Germany) equipped with a ball-on-three-balls (B3B) fixture, as described in a previously established method (11). In this configuration, each specimen was positioned within the B3B fixture between three loading balls located on the upper (compressive) side and one supporting ball on the lower (tensile) side. The testing procedure for the B3B method is illustrated in Figure 2.

The steel balls had equal radii (Rb = 4 mm). The B3B fixture was carefully aligned with the centre of the opposing head of the testing machine to ensure proper loading and specimen fracture. The support guide was then removed to allow free rolling of the loading balls, and a compressive load was applied using a 2 kN load cell at a crosshead speed of 0.5 mm/min until specimen fracture occurred. The biaxial flexural strength σ (MPa) was calculated as the maximum stress at fracture established on the tensile side of the specimen using the following equation:

(7) $$\sigma =\delta \frac{F_{max}}{t^{2}}$$

Here, F_max_ is the fracture force, t is the specimen thickness, and δ is a geometrical correction factor determined using three independent variables: the dimensional ratio, the support radius (R_a_), and the Poisson’s ratio (ν) of the tested material. The support radius (R_a_) can be calculated as R_a_ = (2√3 Rb)/3, where Rb is the radius of the supporting balls. Additional parameters include the radius of the specimen (R) and the thickness-to-specimen-radius ratio (t/R). The Poisson’s ratio (ν) is equal to 0.3 for the dental composite material under testing [19,20]. The value of the geometrical correction factor (δ) was calculated using the provided equation:

(8) $$\delta =0.323308+\frac{\left[\left[\left(1.30843+1.44301v\right)\right]\left[1.78428-3.15347{\left(\frac{t}{R_{a}}\right)}^{2}+6.67919{\left(\frac{t}{R_{a}}\right)}^{2}-4.62603{\left(\frac{t}{R_{a}}\right)}^{3}\right]\right]}{\left[1+1.71955\left(\frac{t}{R_{a}}\right)\right]}$$

### 2.5. Weibull Modulus

To evaluate the reliability of the biaxial flexural strength of the tested 3D-printed resins subjected to different post-curing units, the Weibull analysis was performed to determine the Weibull modulus (m). The Weibull distribution was calculated from the following equation [21]:

(9) $$P_{f}\left(\sigma \;\right)=1-exp\left[-{\left(\;\frac{\sigma }{\sigma 0}\;\right)}^{m}\right]$$

Here, Pf (σ) represents the probability of failure at a given flexural strength, σ is the fracture strength, σ0 is the characteristic strength corresponding to 63.2% probability of failure, and m is the Weibull modulus. The CI 95% confidence intervals (upper and lower limits) of both σ0 and m were calculated in accordance with DIN ENV 843–5:2007 [22].

### 2.6. Martens Hardness and Indentation Modulus (H_M_ and E_IT_)

For the Martens parameter tests, 25 disc-shaped specimens (n = 5 per group) with a diameter of 15 mm and a thickness of 2 mm were prepared. All specimens were manually polished using silicon carbide abrasive papers up to 1200 grit (Buehler, Lake Bluff, USA) to remove any remaining flanges and to ensure a standardised, smooth surface. After polishing, the specimens were stored under dry conditions for 24 h before testing.

The Martens hardness test was conducted using a hardness testing machine (Zwick/Roell ZHU2.5, Zwick GmbH & Co., Ulm, Germany) equipped with a Vickers indenter and operated through testXpert V12.6 Master software. Each specimen was centrally positioned beneath the indenter tip. A load was applied at a rate of 5 N/s until reaching a maximum load of 10 N, with a crosshead speed of 1 mm/min and a dwell time of 30 s. The initial approach rate of the indenter was set to 100 mm/min, followed by a reduced approach rate of 40 mm/min for final contact. After initial contact, the indenter was positioned 40 µm from the specimen surface, and a fixed distance of 13 mm was maintained between the measuring head and the upper specimen surface at the start of each test.

Each specimen was subjected to five controlled random indentations on the same surface, with a spacing of 1 mm between indentations. The indentation depth and the corresponding load during loading and unloading were automatically recorded, generating load–displacement curves. Martens hardness (H_M_) and indentation modulus (E_IT_) values were automatically calculated and expressed in N/mm^2^ and kN/mm^2^, respectively, according to ISO 14577-4:2016 [23], using the following equations:

(10) $$H_{M}=\frac{F}{A_{s\;\;}\left(h\right)}=\;\frac{F}{26.43^{x}h^{2}}$$

(11) $$E_{IT}=1-v_{s}^{2}\times \;(\frac{1}{E\gamma }-\frac{\left(1-v_{i}^{2}\right)}{Ei})$$

where *H_M_* is expressed in N/mm^2^, *F* is the applied load in Newtons (N), and *As(h)* is the surface area of the indenter at a specified distance h from its tip, in mm^2^. *Eγ* represents the reduced modulus of the indentation contact, and *Ei* is the elastic modulus of the indenter. The Poisson’s ratios for the specimen (*v_s_*) and the indenter (*v_i_*) were 0.35 and 0.30, respectively [24,25].

### 2.7. Statistical Analysis

Statistical analysis was performed using IBM SPSS Statistics version 29 (IBM, New York, NY, USA). Data distribution was evaluated by the Shapiro–Wilk test, and the homogeneity of the variance was evaluated by the Laven’s test. All data were normally distributed; however, the assumption of homogeneity of the variance was violated for the water solubility (Wsl), Marten’s hardness and indentation modulus. Accordingly, one-way ANOVA was performed for the biaxial flexural strength and water sorption tests (Wsp), followed by the Tukey post hoc test. For the Martens hardness, water solubility and indentation modulus, Welch one-way ANOVA was performed, followed by Games-Howell post hoc analysis. A Pearson correlation test was performed to evaluate the relationship between water sorption and water solubility values. The significance level was set at α = 0.05.

## 3. Results

### 3.1. Water Sorption and Solubility

**Figure 3:**
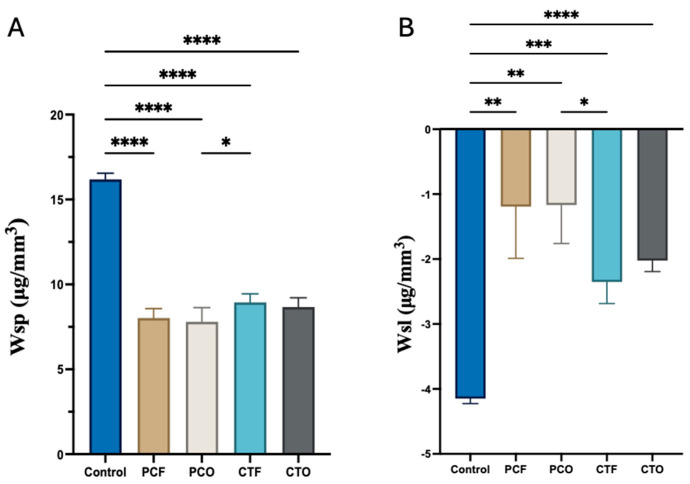
Graph illustrating the water sorption and solubility values of the tested groups. (**A**) Sorption values. (**B**) Solubility values. * *p* < 0.05 (statistically significant); ** *p* < 0.01 (very statistically significant); *** *p* < 0.001 (highly statistically significant); **** *p* < 0.0001 (extremely statistically significant).

The results of the mean and standard deviation values of the water sorption and solubility are presented in Table 4 and Figure 3. For the water sorption test, one-way ANOVA exhibited a significant difference between the tested groups (*p* < 0.001). The highest value of the water sorption was found in the control group (16.2 μg/mm^3^), which was significantly higher than all 3D-printed resin groups (*p* < 0.001). In contrast, the lowest value was recorded in the PCO group (7.7 μg/mm^3^), which was significantly different from the CTF (*p* = 0.039). Importantly, both PC and CT resins showed no statistically significant difference when different postcuring units were used (PCF vs. PCO, CTF vs. CTO) (*p* > 0.05).

In contrast, Welch’s one-way ANOVA showed a statistically significant difference between the groups (*p* < 0.001). As for the water sorption results, the control group (−4.1 μg/mm^3^) showed the highest value of the water solubility, which was significantly different from all 3D-printed groups (*p* ≥ 0.005), followed by CTF, CTO and PCF, and the lowest was recorded for the PCO group (−1.1 μg/mm^3^), which was only significantly different from the CTF group, and both PC (PCF vs. PCO) and CT (CTF vs. CTO) resins showed no statistically significant difference when different post-curing units were used (*p* > 0.05). A strong negative correlation existed between water sorption and water solubility (r = –0.836, *p* < 0.001).

**Figure 4:**
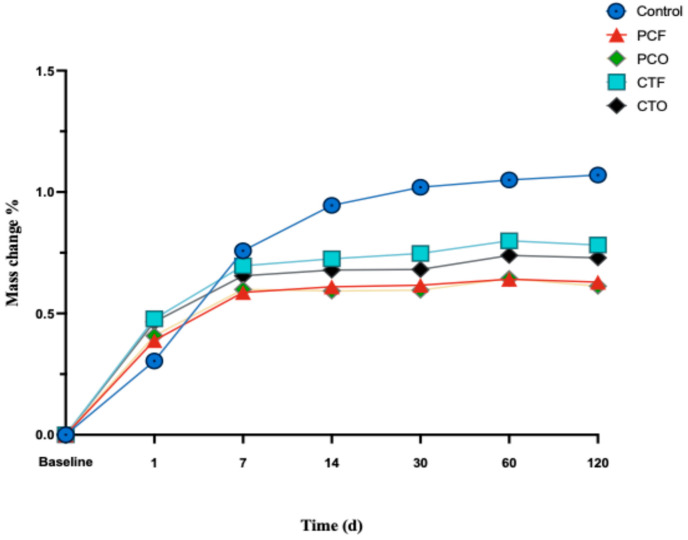
Mass change (%) of the tested groups over 90 days of immersion in distilled water.

All study groups showed a change in mass shortly after 24 h of immersion in distilled water. Initially, the 3D-printed resin groups demonstrated a greater increase in mass compared to the control group at 24 h. However, at the subsequent immersion periods, the control group showed a higher mass increase over the 3D-printed resins. Among the 3D-printed groups, PC resin groups (PCF, PCO) showed slightly lower mass change than the CT resin (CTF, CTO) (Figure 4).

### 3.2. Biaxial Flexural Strength

**Figure 5:**
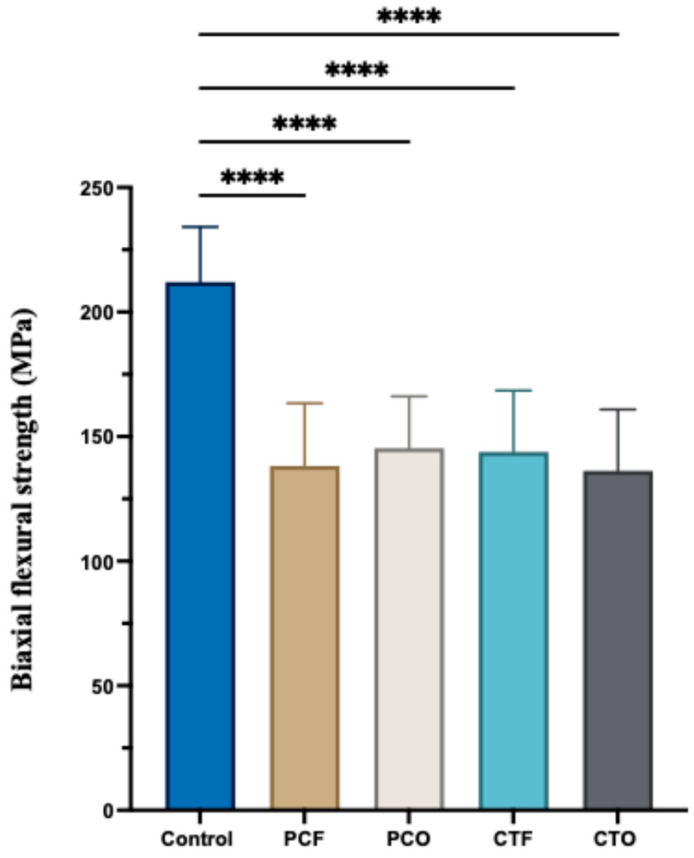
Biaxial flexural strength (MPa) of the tested groups, **** *p* < 0.0001.

The mean and standard deviation values of the biaxial flexural strength are presented in Table 5 and illustrated in Figure 5. One-way ANOVA revealed a statistically significant difference between the tested groups (*p* < 0.001). The control group (212.1 MPa) exhibited the highest value of biaxial flexural strength, which was statistically significant compared to all 3D-printed resin groups (*p* < 0.001), whereas the CTO group (136.2 MPa) was the lowest. Among the 3D-printed resin groups, no statistically significant difference was found when different postcuring units were used either for the PC or CT resin groups (*p* > 0.05).

### 3.3. Weibull Modulus

**Figure 6:**
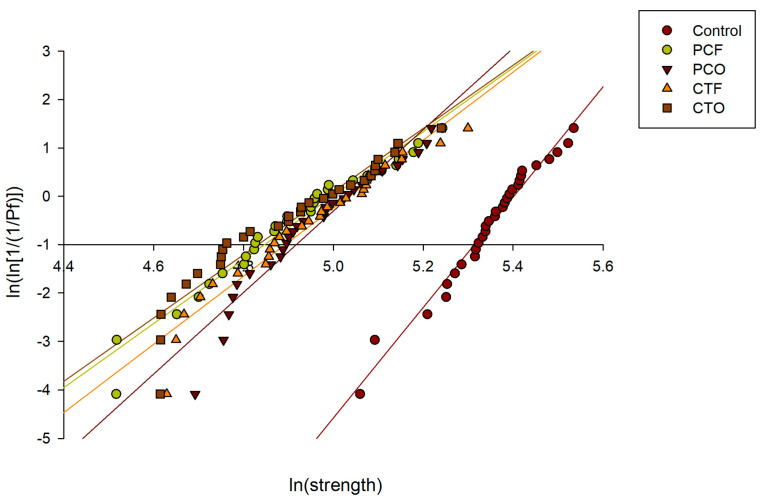
Weibull probability plots of the tested groups.

The values of the *σ*_0_, m and correlation coefficients (R2) obtained from the biaxial flexural strength test are presented in Table 6. The strength data of all tested groups exhibited an excellent linear fit on the Weibull plots, with R^2^ ranging from 0.91 to 0.97 (Figure 6). The control group showed the highest characteristic strength (σ_0_ = 221.7 MPa) and a Weibull modulus (m = 11.3), indicating low variability in strength. Among the 3D-printed resins, for the PC resin, the PCO group demonstrated a higher Weibull modulus (m = 8.4) than the PCF group (m = 6.6), with characteristic strengths of 152.9 MPa and 139.7 MPa, respectively. For the CT resin, the CTF group exhibited a higher Weibull modulus (m = 7.0) than the CTO group (m = 6.5), with characteristic strengths of 153.6 MPa and 146.2 MPa, respectively.

### 3.4. Martens Hardness and Indentation Modulus (H_M_ and E_IT_)

**Figure 7:**
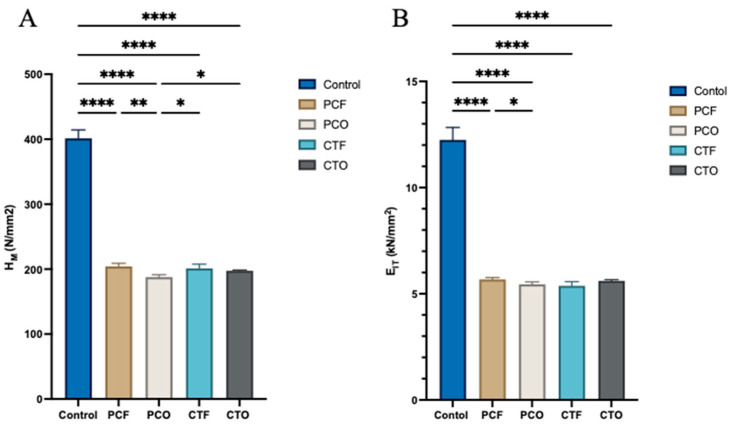
Graph illustrating the Martens hardness (H_M_) and Indentation modulus (E_IT_) values of the tested groups. (**A**) Martens hardness. (**B**) Indentation modulus. * *p* < 0.05 (statistically significant); ** *p* < 0.01 (very statistically significant); *** *p* < 0.001 (highly statistically significant); **** *p* < 0.0001 (extremely statistically significant).

The mean and standard deviation values of Martens hardness and indentation modulus for the tested groups are presented in Table 7 and Figure 7. For the Martens hardness test, Welch’s one-way ANOVA revealed a statistically significant difference between the groups (*p* < 0.001). The control group exhibited the highest value (401.4), which was significantly greater than all 3D-printed resin groups (*p* < 0.001). Among the 3D-printed resins, the PCO group showed the lowest value (187.7), which was significantly lower than the PCF group (204.1; *p* = 0.003) and both CT resin groups (*p* ≤ 0.035). No statistically significant difference was observed between the CT resin groups (CTF, CTO; *p* = 0.750).

Welch’s one-way ANOVA for the indentation modulus also showed a statistically significant difference among the tested groups (*p* < 0.001). Similar to the Martens hardness results, the control group exhibited the highest indentation modulus (12.26), which was significantly higher than all 3D-printed resin groups (*p* < 0.001). Among the 3D-printed resins, the PC resin showed a significant difference between the PCF (5.67) and PCO (5.44) groups (*p* = 0.047). No statistically significant difference was found between the CT resin groups (CTF and CTO) when different post-curing units were used (*p* > 0.05).

## 4. Discussion

This laboratory study evaluated the effect of different post-curing units on the physical and mechanical properties of 3D-printed resins used for the fabrication of permanent crowns and compared them with a conventional resin composite. The findings revealed that the control group exhibited superior mechanical properties compared to the 3D-printed resins, along with higher water sorption and solubility values. Furthermore, significant differences in microhardness were observed between the post-curing units. Therefore, the null hypothesis stating that there would be no significant differences between the control and 3D-printed groups or between the post-curing units was partially rejected.

For the 3D-printed resins used in this study, the manufacturers did not disclose the specific type or concentration of photoinitiators, and each recommended distinct 3D-printing and post-curing protocols. The PC resin was printed using SLA technology at 405 nm and post-cured with the Formcure device (Formlabs, Somerville, MA, USA), which operates at the same wavelength. In contrast, the CT resin was fabricated using DLP technology at 385 nm and post-cured with the Otoflash G171 (NK-Optik GmbH, Baierbrunn, Germany), which emits a broader wavelength spectrum (280–700 nm) to promote complete polymerisation. Because photoinitiator composition and activation wavelength influence polymerisation efficiency, post-curing conditions can markedly affect material performance [2,12,26]. Hence, evaluating different post-curing devices is essential for understanding their influence on the physical and mechanical properties of 3D-printed dental resins.

### 4.1. Water Sorption and Solubility

A characteristic feature of polymer-based materials, particularly resin composites, is their tendency to absorb water over time. This water uptake can lead to softening, chemical degradation, and elution of unreacted monomers, generating internal stresses and promoting crack formation, which ultimately compromise the material’s mechanical properties [27]. Accordingly, water sorption and solubility tests are well-established methods for assessing the ability of resin-based materials to withstand exposure to oral fluids [28]. Therefore, polymer-based materials should exhibit low water sorption and solubility to preserve their structural integrity and long-term performance.

In this study, our results indicate that all tested groups met the property requirements specified by the ISO 4049 standard. After 90 days of water storage, the values for water sorption and solubility remained below 40 µg/mm^3^ [28]. The 90-day storage in water was selected as repeated measurements showed no further change in specimen mass, indicating that equilibrium had been achieved. Moreover, our findings indicate that both the control group and the 3D-printed resins used in this study, when post-cured in different post-curing units, can handle a moist environment properly. In our study, the control group exhibited the highest values, 16.1 and −4.1 µg/mm^3^, for both water sorption and solubility, respectively, which were significantly higher than those of all 3D-printed resin groups. Nonetheless, water sorption has been reported to correlate negatively with filler content, as increasing the filler weight within a composite reduces the volume of hydrophilic resin matrix available to absorb water [29]. However, the results of this study contradict this relationship, as the control group, despite having the highest filler loading (76.5 wt%), displayed higher water sorption than the 3D-printed resins, which contained only 30–50 wt% fillers. The 3D-printed materials, with lower filler content, showed significantly lower water sorption values (7.1–8.9 µg/mm^3^). This may be related to the resin chemistry, as AUDMA contains polar secondary amine groups (-NH-) within its urethane linkages, which can act as hydrogen-bonding sites for water molecules and thereby increase water uptake [30]. In contrast, the 3D-printed resins (Crowntec and Permanent Crown) are Bis-EMA-based, which is characterised by its high hydrophobicity, which reduces retention of water within the polymer network [31]. Additionally, the layer-by-layer polymerisation and subsequent post-curing of 3D-printed resins results in a higher degree of conversion and cross-link density than that achieved in conventionally cured Filtek™ Universal composite [13,15]. Although these differences were statistically significant, all values remained within the ISO standard limits [28], suggesting limited clinical relevance.

In a previous study by González-Alenda et al. [32], Filtek™ Universal Composite (control group) exhibited a water sorption value of 18.0 µg/mm^3^ after 7 days of immersion in distilled water, which is slightly higher than the value recorded in this study (16.2 µg/mm^3^) after 90 days of water storage, which might be attributed to the specimen preparation. Nonetheless, this similarity in the finding may indicate that the control group maintains a constant water sorption and exhibits long-term hydrolytic stability. Meanwhile, for the 3D-printed resins, no significant differences were observed between post-curing units within the same material, all curing protocols used in this study produced comparable polymerisation and hydrolytic stability. This suggests that the light spectra and energy delivered by both units were sufficient to effectively activate the material’s photoinitiators, resulting in consistent physical properties regardless of the specific device.

Among the 3D-printed resins used in this study, the CT resin showed a water sorption value of 8.9 and 8.6 µg/mm^3^, in Formcure and Otoflah G171 postcuring devices, respectively, which is slightly lower than the range of 10.6–12.6 µg/mm^3^ of water sorption reported in the literature [33,34]. This difference may be attributed to variations in postcuring and cleaning protocols. Specifically, the extended postcuring time (40 min vs. 20 min) and the use of 98.8% ethanol for washing before postcuring may have enhanced polymer conversion and reduced residual monomer content. In contrast, previous studies used a variety of washing solutions, including InovaPrint™ Wash, 96% ethanol, or 90% denatured ethanol [34].

For the PC resin, no previous data were available for direct comparison; however, its lower sorption (8.0 and 7.7 µg/mm^3^ in Formcure and Otoflahs G171, respectively) suggests a more hydrophobic composition or higher cross-link density under identical curing conditions. Both materials exhibited water sorption values well below the ISO 4049 limit (40 µg/mm^3^), confirming their excellent hydrolytic stability. When comparing PC and CT resins, the only statistically significant difference was observed between PCO and CTF; however, the magnitude of this difference was negligible, confirming the overall consistency of the tested properties across both curing protocols. This suggests comparable polymerisation efficiency between the two materials under the tested post-curing conditions.

All tested study groups demonstrated water solubility values below the ISO 4049 standard of 7.5 µg/mm^3^ [28], indicating the adequate chemical stability of the tested materials after 90 days of immersion in distilled water. Interestingly, all groups exhibited negative solubility values, a phenomenon that has also been reported in previous studies [29]. The negative values likely result from incomplete dehydration during the desiccation phase, leaving residual bound water within the polymer matrix. Alternatively, they may indicate limited elution of unreacted monomers or hydrophilic components, suggesting that water uptake exceeded material loss from the resin matrix.

The control group retained significantly more water than all 3D-printed resin groups, a finding supported by a strong negative correlation in the Pearson correlation test (r = –0.836, *p* < 0.001) and confirmed by the higher mass change percentage observed in this study (Figure 4). No statistically significant differences were found within or between all 3D-printed resin groups, indicating that both curing protocols led to comparable effects on water solubility.

### 4.2. Biaxial Flexural Strength and Weibull Modulus

The biaxial flexural strength (BFS) test is an essential mechanical property for evaluating the durability of restorative materials under clinical conditions, as it reflects the material’s resistance to failure under the combined tensile and compressive stresses typical of masticatory forces in the oral environment [35]. The BFS test is traditionally used for brittle materials such as dental ceramics, but it has also been increasingly applied to dental composites, as it provides comparable results with less variability than uniaxial flexural strength testing [36]. The ball-on-three-balls (B3B) method was used in this study to measure BFS because it offers several advantages over other testing methods, including insensitivity to minor surface irregularities and ease of specimen preparation. In addition, no edge adjustments are required, as the applied force is directed centrally on the specimen, unlike the uniaxial flexural test [37,38], which allows for accurately measuring the actual force required for the fracture of the specimen. Based on the results of the current study, all tested groups exceeded the >50 MPa threshold for single-unit restorations recommended by the ISO 10477 standard for polymer-based materials [16].

The result of the BFS of the control group is comparable with the result of the flexural strength reported in the previous study [39]. In the current study, the control group exhibited significantly higher BFS (212.1 MPa) compared with all tested 3D-printed resins (136.2–145.3 MPa). This difference is due to the filler content, as the control group contains 76.5 wt% inorganic fillers. In contrast, the 3D-printed resins contain only 30–50 wt%, as reported in a previous study, which found that increasing the filler content also increases the mechanical properties of the resin composite [40]. The reduced filler content in the 3D-printed resin is necessary for the rheological properties of the dental resin required during 3D-printing [35].

On the other hand, flexural strength has been positively correlated with the degree of conversion of the monomer into polymer within the resin composite. This process is influenced by an optimal wavelength that effectively interacts with the photoinitiators present in the resin composite, leading to an enhanced degree of conversion [41]. For the 3D-printed resins used in this study, the influence of different post-curing devices on completing the polymerisation process and, consequently, on the biaxial flexural strength was investigated. Lassila et al. [41] investigated the effect of different post-curing units on the flexural strength and surface wear resistance of two 3D-printed resins. They concluded that variations in post-curing conditions significantly influenced these properties in the tested materials. However, in the current study, no significant differences were observed between the Formcure and Otoflash G171 devices in terms of BFS, either within or between the PC resin and CT resin groups, suggesting comparable curing efficiency for both materials. This discrepancy likely stems from differences in the chemical composition of the resins and the specific light-curing technologies employed. This suggests that the impact of post-curing protocols is material-specific, highlighting the necessity of evaluating each resin independently rather than extrapolating a universal response across curing configurations.

Weibull analysis is practical to employ to evaluate the reliability and predictability of the mechanical performance of brittle materials such as dental composites and ceramics. Unlike mean strength values, it accounts for the variability arising from intrinsic flaws and microstructural imperfections. The Weibull modulus (m) is a critical parameter that quantifies the variability in strength data of the material; a higher modulus suggests greater reliability and lower data scatter in its strength, which is a desirable property for clinical applications [8]. The characteristic strength (σ_0_) corresponds to the stress level at which 63.2% of specimens are predicted to fail, providing a more dependable measure of strength than the mean value [35]. The control group exhibited the highest Weibull modulus m = 11.3 and σ0 = 221.7 MPa, suggesting that it has higher reliability and more consistent strength compared to all 3D-printed resin groups tested in the current study. Among the 3D-printed groups, the PC resin post-cured with the Otoflash G171 (PCO) exhibited a higher Weibull modulus (m = 8.4, σ_0_ = 152.9 MPa) compared to the Formcure-treated group (PCF) (m = 6.6, σ_0_ = 146.9 MPa). This suggests that post-curing the PC resin with the Otoflash G171 resulted in a more consistent and reliable biaxial flexural strength (BFS). Conversely, for the CT resin, specimens post-cured using the Formcure device (CTF) demonstrated a higher Weibull modulus (m = 7.0, σ_0_ = 153.6 MPa) than those cured with the Otoflash G171(CTO) (m = 6.5, σ_0_ = 146.2 MPa), indicating that the Formcure device provided a more uniform and dependable BFS. This material-dependent response may be explained by differences in the printing wavelength and post-curing spectrum: the PC resin was printed at 405 nm and the CT resin at 385 nm, whereas Formcure delivered fixed 405 nm light with elevated temperature and Otoflash provided a broader flash-curing spectrum. Accordingly, each resin may have responded optimally to the post-curing condition that more closely matched its photoinitiator system and curing kinetics [42]. Another possible explanation is the photoinitiator type in each resin, since different initiator systems exhibit different spectral sensitivity and radical-generating efficiency, potentially leading to resin-specific differences in post-curing response [43].

### 4.3. Martens Hardness and Indentation Modulus

Traditionally, hardness evaluation of dental materials has relied on Knoop or Vickers tests; however, these methods have certain limitations, such as sensitivity to the applied load, dwell time, and operator variability. In contrast, the Martens hardness (HM) test has been introduced as a more advanced and reliable technique, eliminating the limitations associated with the traditional hardness tests, particularly well-suited for assessing the combined elastic and plastic behaviour of dental materials [9].

The hardness of dental composites can be influenced by different factors other than the degree of conversion of the resin composite, including the inorganic filler content [44]. In this study, the Filtek™ Universal Restorative (control group) exhibited the highest Martens hardness value, which was significantly greater than those of all 3D-printed resins tested. This difference can be attributed primarily to the disparity in filler content, as the control composite contains approximately 76.5 wt% inorganic fillers, whereas the 3D-printed resins contain considerably lower amounts, ranging between 30 and 50 wt%, which aligns with the results of a previous study, which indicated that higher inorganic filler content enhanced the hardness of the resin composite [45].

In the 3D-printing of photopolymer materials, the process begins with partial layer-by-layer polymerisation of the printed object, which must then be followed by post-polymerisation to complete the polymerisation cycle. In this study, the composition and concentration of the photoinitiator system used in both 3D-resins were not disclosed by the manufacturer. Both the type and concentration of photoinitiators in the resin matrix can affect surface microhardness [46], while the light source and exposure duration also influence the final hardness of dental composites [47]. Therefore, given that both the photoinitiator system and the curing conditions can influence the polymerisation and hardness of 3D-printed resins, this study further evaluated how different post-curing units affect the mechanical properties of two commercial resin types.

In this study, two different post-curing units were used: the Formcure and the Otoflash G171, each employing different light sources and curing technologies. Unlike the BFS results, the PC resin exhibited significantly higher hardness values when processed with the Formcure device (PCF group) compared to the Otoflash G171 (PCO group). This divergence likely stems from the fundamental difference between bulk properties and surface-related properties. Surface hardness is highly sensitive to the immediate curing environment; as established by Dantagnan et al. [48] and Reymus et al. [15], both device type and thermal parameters significantly influence the degree of conversion of the 3D-printed resins. The extended duration (40 min) and elevated temperature (60 °C) of the Formcure unit likely accelerated polymerisation and enhanced free-radical diffusion, leading to greater surface cross-linking [2]. In contrast, the shorter duration (~7 min) and limited thermal control of the Otoflash G171 may result in lower surface polymerisation efficiency, whereas bulk properties like BFS remain stable once a general polymerisation saturation point is reached. For the CT resin, both post-curing groups (CTF and CTO) showed significantly higher hardness than PCO, indicating improved polymerisation under the tested conditions. Although the difference between PCF and PCO was statistically significant, its magnitude was small, and no significant difference was observed in biaxial flexural strength. Accordingly, this hardness difference is unlikely to have a meaningful impact on wear resistance or long-term structural performance. The absence of significant differences within the CT resin groups further suggests that the effect of post-curing conditions is material dependent.

One of the main advantages of the Martens hardness test is that it can measure the elastic property of materials by providing the indentation modulus (E_IT_) of the tested specimen in a single measurement [9], allowing for the determination of the elastic deformation of dental materials. Similarly to the Martens hardness, the highest value of the EIT was observed in the control group, which was significantly higher than that of the 3D-printed resins tested in the current study. This difference can be attributed to the higher filler content of the conventional resin composite compared to the 3D-printed resins, consistent with previous findings [49].

Within the 3D-printed resins, the E_IT_ showed a trend similar to that of Martens hardness (H_M_). A statistically significant difference was observed only between the PC resin groups (*p* = 0.047), with the Formcure group (PCF) exhibiting a slightly higher EIT (5.6 kN/mm^2^) compared to the Otoflash group (PCO, 5.4 kN/mm^2^). This can be attributed to the extended post-curing duration and controlled thermal environment of the Formcure device, which facilitates more extensive polymerisation and cross-linking than the shorter, flash-based curing of the Otoflash G171. In contrast, for the CT resin, no significant difference in E_IT_ was found between the two post-curing units, further confirming that the influence of the used post-curing conditions on elastic properties is material dependent.

The findings of this study should be interpreted with caution due to its in vitro design, which may not fully replicate clinical conditions. Moreover, this time, only two 3D-printed resins were compared; evaluating additional formulations could provide a more comprehensive understanding of their mechanical and physical behaviour. In addition, distilled water was used for the evaluation of water sorption and solubility in accordance with ISO 4049 to ensure standardised testing conditions. However, distilled water does not fully replicate the complexity of the oral environment; therefore, the use of artificial saliva may yield different results and provide greater clinical relevance. Future studies should consider employing artificial saliva to better simulate intraoral conditions. Finally, this study evaluated only two 3D-printable resins and two post-curing units under specific conditions. Given the variability in composition and formulation among commercially available 3D-printed resins, the findings of this study cannot be generalised to all materials. In addition, evaluating other curing devices and processing parameters may yield different outcomes [50]. Therefore, further research involving a broader range of materials, curing systems, and environmental conditions is recommended to understand better the influence of post-curing on the properties of 3D-printed resins.

## 5. Conclusions

This laboratory study assessed the influence of two post-curing units with specific parameters on the water sorption and solubility, Martens hardness, indentation modulus, and biaxial flexural strength of two 3D-printed resins for permanent crown fabrication, compared and contrasted with a conventional resin composite. Within the study’s limitations, the following conclusions can be drawn:

- The conventional resin composite exhibited higher water sorption than both 3D-printed resins; however, post-curing conditions produced comparable results for the PC and CT materials.
- All groups showed negative water solubility values, with the control group retaining more water than the 3D-printed resins.
- The control group demonstrated the highest Martens hardness and indentation modulus. A statistically significant difference between post-curing units was observed only for the PC resin, while no significant difference was observed for the CT resin.
- The biaxial flexural strength and Weibull modulus were highest in the control group, with no significant differences detected between or within the 3D-printed resins. While biaxial flexural strength remained consistent across all 3D-printed groups, the opposing trends in Weibull modulus for PC and CT resins further suggest that these materials do not respond uniformly to postcuring protocols.

## 6. Clinical Significance

While minor variations in hardness and modulus were observed between curing units, these differences were not clinically significant. For both resin materials, the post-curing protocols resulted in comparable physico-mechanical properties under the tested conditions, indicating similar performance.

## Figures and Tables

**Table 1:** Materials used in the study.

Material	Manufacturer	Composition	Lot.
Filtek Universal resin composite^TM^ Shade: A2 (Control)	3M UK	Organic content: AUDMA, AFM, diurethane-DMA, and 1,12-dodecane-DMA. Inorganic content: combination of a non-agglomerated/non-aggregated 20 nm silica filler, a non-agglomerated/non-aggregated 4 to 11 nm zirconia filler, an aggregated zirconia/silica cluster filler (comprising 20 nm silica and 4 to 11 nm zirconia particles), and a ytterbium trifluoride filler consisting of agglomerated 100 nm particles. The inorganic filler loading is about 76.5% by weight (58.4% by volume).	3M-6555A2
CrownTec^TM^ (3D resin) Shade: A2 (CT)	SAREMCO Dental AG Gewerbestrasse 4 CH-9445 Rebstein/Switzerland	Esterification products of 4,4′-isopropylidiphenol, ethoxylated and 2-methylprop-2enoic acid, silanized dental glass, Pyrogenic silica, initiators. Total content of inorganic fillers (particle size 0.7 μm) is 30–50% by mass.	8052
Permanent Crown^TM^ (3D resin) Shade: A2 (PC)	Formlabs Funkhaus Berlin (Block A, 2. Etage) Nalepastraße 18, 12459 Berlin, Germany	Esterification products of ethoxylated 4,4′-isopropylidiphenol and 2-methylprop-2-enoic acid. - Silanized dental glass. Methylbenzoyl diphenyl format: diphenyl(2,4,6-trimethylbenzoil) phosphine oxide. Total content of inorganic fillers (particle size 0.7 µm): 30–50 wt%	600165

AUDMA, Aromatic Urethane dimethacrylate; AFM (addition fragmentation monomers); DMA, dimethacrylate.

**Table 2:** The 3D printers used for each 3D-printed resin.

3D Resin	3D-Printer	Manufacturer	Printer Specification	Printing Technology	Slicing Software
Permanent Crown^TM^	Form 3B+	Formlabs Inc., USA	385 nm (high power UV LED) or 405 nm	SLA	PreForm
CrownTec^TM^	Asiga Max	ASIGA, Australia	405 nm wavelength 250 mW power 85 µm laser spot	DLP	Composer

**Table 3:** Postcuring parameters used for each 3D-printed resin used in this study.

Device	Manufacturer	Light Source	Wavelength	Post-Curing Parameter
**Otoflash G171** **Code (O)**	NK-Optik GmbH	2 flashbulbs at 100 w Adjustable flashes number	280–700 nm maximum between 400 and 500 nm	2000 Flashes on each side
**Form Cure** **Code (F)**	Formlabs	13 multi-directional LEDs Adjustable time and temperature	405 nm	20 min at 60 °C on each side

**Table 4:** Means and SD of the water sorption and water solubility at 90 days of immersion.

Materials	Sorption (μg/mm^3^)	Solubility (μg/mm^3^)	Sorption %	Solubility %
Control	16.2 (0.3) ^a^	−4.1 (0.1) ^a^	0.83 (0.02)	−0.24 (0.04)
PCF	8.0 (0.5) ^b,c^	−1.1 (0.7) ^b,c^	0.55 (0.03)	−0.04 (0.13)
PCO	7.7 (0.8) ^b^	−1.1 (0.5) ^b^	0.53 (0.06)	−0.08 (0.04)
CTF	8.9 (0.5) ^c^	−2.3 (0.3) ^c^	0.62 (0.03)	−0.16 (0.02)
CTO	8.6 (0.5) ^b,c^	−2.0 (0.1) ^b,c^	0.59 (0.04)	−0.14 (0.01)

Different superscript letters indicate statistically significant differences within the same column (*p* < 0.05). Pearson’s correlation analysis revealed a strong negative correlation between water sorption and solubility (r = −0.836, *p* < 0.001).

**Table 5:** Means and (SD) of the Biaxial flexural strength (MPa) of the tested groups.

Groups	Biaxial Flexural Strength
Control	212.1 ± (22.1) ^a^
PCF	138.2 ± (25.1) ^b^
PCO	145.3 ± (20.8) ^b^
CTF	143.8 ± (24.6) ^b^
CTO	136.2 ± (24.6) ^b^

Different superscript letters indicate statistically significant differences (*p* < 0.05).

**Table 6:** Characteristic strength σ0 (MPa), Weibull modulus (m), and coefficient of determination (R2) of the tested groups.

Groups	Characteristic Strength σ_0_ (MPa) [95% CI]	Weibull Modulus (*m*) [95% CI]	Coefficient of Determination (R^2^)
Control	221.7 (215.3, 228.3)	11.3 (8.5, 13.9)	0.97
PCF	146.9 (139.7, 154.6)	6.6 (4.9, 8.1)	0.96
PCO	152.9 (146.9, 159.1)	8.4 (6.3, 10.2)	0.93
CTF	153.6 (146.5, 161.2)	7.0 (5.2, 8.5)	0.95
CTO	146.2 (138.9, 153.9)	6.5 (4.8, 7.9)	0.91

**Table 7:** Means and SD of the Martens hardness (H_M_) (N/mm^2^) and indentation modulus (E_IT_) (kN/mm^2^) of the tested groups.

Materials	Martens Hardness (N/mm^2^) (HM) Mean ± Standard Deviation	Indentation Modulus (kN/mm^2^) (Mean ± Standard Deviation
Control	401.4 (12.9) ^a^	12.24 (0.2) ^a^
PCF	204.1 (4.8) ^b^	5.67 (0.03) ^b^
PCO	187.7 (3.5) ^c^	5.44 (0.1) ^c^
CTF	201.1 (6.5) ^b^	5.37 (0.1) ^b,c^
CTO	197.5 (0.9) ^b^	5.61(0.2) ^b,c^

Different superscript letters within the same column indicate statistically significant differences (*p* < 0.05).

## Data Availability

The original contributions presented in the study are included in the article. Further inquiries can be directed to the corresponding author.

## Abbreviations

The following abbreviations are used in this manuscript:

B3B	Ball on 3 balls
BPF	Powder bed fusion
CAD/CAM	Computer aided design/computer aided manufacturing
CTF	CrownTec resin postcured with Formcure device
CTO	CrownTec resin postcured with Otoflash G171 device
DLP	Digital light processing
E_IT_	Indentation modulus
FDM	Fused deposition modeling
H_M_	Martens hardness
PCF	Permanent Crown resin postcured with Formcure device
PCO	Permanent Crown resin postcured with Otoflash G171 device
SLA	Steriolithography
SLM	Selective laser melting
SLS	Selective laser sintering
STL	Standard tessellation language
